# All nonhomologous chromosomes and rearrangements in *Saccharum officinarum* × *Saccharum spontaneum* allopolyploids identified by oligo-based painting

**DOI:** 10.3389/fpls.2023.1176914

**Published:** 2023-10-06

**Authors:** Jin Chai, Li Xue, Jiawei Lei, Wei Yao, Muqing Zhang, Zuhu Deng, Fan Yu

**Affiliations:** ^1^ State Key Laboratory for Conservation and Utilization of Subtropical Agro-bioresources, Guangxi University, Nanning, China; ^2^ Guangxi Key Laboratory for Sugarcane Biology, Guangxi University, Nanning, China; ^3^ National Engineering Research Center for Sugarcane, Fujian Agriculture and Forestry University, Fuzhou, Fujian, China; ^4^ Key Laboratory of Sugarcane Biology and Genetic Breeding, Ministry of Agriculture and Rural Affairs, Fujian Agriculture and Forestry University, Fuzhou, China

**Keywords:** sugarcane, interspecific hybridization, nobilization, chromosomal inheritance, oligo-FISH

## Abstract

Modern sugarcane cultivars (*Saccharum* spp., 2*n* = 100~120) are complex polyploids primarily derived from interspecific hybridization between *S. officinarum* and *S. spontaneum*. Nobilization is the theory of utilizing wild germplasm in sugarcane breeding, and is the foundation for utilizing *S. spontaneum* for stress resistance. However, the exact chromosomal transmission remains elusive due to a lack of chromosome-specific markers. Here, we applied chromosome-specific oligonucleotide (oligo)-based probes for identifying chromosomes 1-10 of the F_1_ hybrids between *S. officinarum* and *S. spontaneum*. Then, *S. spontaneum*-specific repetitive DNA probes were used to distinguish *S. spontaneum* in these hybrids. This oligo- fluorescence *in situ* hybridization (FISH) system proved to be an efficient tool for revealing individual chromosomal inheritance during nobilization. We discovered the complete doubling of *S. officinarum-*derived chromosomes in most F_1_ hybrids. Notably, we also found defective *S. officinarum*-derived chromosome doubling in the F_1_ hybrid Yacheng75-4191, which exhibited 1.5n transmission for all nonhomologous chromosomes. Altogether, these results highlight the presence of variable chromosome transmission in nobilization between *S. officinarum* and *S. spontaneum*, including 1.5n + n and 2n + n. These findings provide robust chromosome markers for in-depth studies into the molecular mechanism underlying chromosome doubling during the nobilization, as well as tracing chromosomal inheritance for sugarcane breeding.

## 1 Introduction

Sugarcane (*Saccharum* spp.), a member of the family *Poaceae*, is a complex heterozygous polyploid plant, exhibiting up to decaploidy or even higher (Grivet and Arruda, 2002; Piperidis and D’Hont, 2020). Sugarcane has been received more attention and is used to produce sugar as well as energy. *S. officinarum* (2*n* = 80 or 81, *x* = 10) exhibits a high sugar content, but poor environmental resistance (Irvine, 1999; Aitken et al., 2006; Piperidis et al., 2010). In contrast, *S. spontaneum* (2*n* = 40 ~ 128, *x* = 8, 9, 10), which is distributed widely from the Mediterranean to the Pacific, is highly stress-resistance (Panje and Babu, 1960). *S. spontaneum* is typically hybridized with *S. officinarum* (Price, 1957; BT, 1972). Thus, sugarcane is an allopolyploid produced by repeated interspecific hybridization. Allopolyploidy, which results in genetic redundancy, is a source of new variation. In sugarcane, such allopolyploidy results in increased drought tolerance, pest resistance, and biomass production etc. (Osborn et al., 2003; Chen, 2007). Despite its economic importance, the complexity of the sugarcane genetic background has limited classical genetic studies (Barnes and Bester, 2000). The high ploidy level of interspecific *Saccharum* hybrids, and the variability of the hybridization environment, further complicate sugarcane genetic research.

The complex genetic background of cultivated sugarcane is the result of multiple interspecific hybridization events, particularly between *S. officinarum* and *S. spontaneum*. The first artificial interspecific hybrids between these two species were created to overcome disease outbreaks and were followed by repeated backcrossing using *S. officinarum* as the recurrent female parent to restore high sucrose content. This process, known as ‘nobilization’, has been central to sugarcane genetic improvement (D'Hont et al., 1996; Ming et al., 2010). Jeswiet has bred a series of excellent parental materials over the past few decades, including the ‘POJ’ series (Heinz, 1987). This strategy combines the stress resistance of *S. spontaneum* with the high yield and sugar content of *S. officinarum* (Jackson, 2005). Grivet et al. found that 15~25% lineages of the sugarcane cultivar R570 were derived from recombination between *S. officinarum* and *S. spontaneum* (Grivet et al., 1996). Recent cytogenetic studies have shed light on the basic chromosome number of *S. spontaneum* and chromosomal inheritance during sugarcane breeding (Cuadrado et al., 2004; Edmé et al., 2006). A variety of chromosomal inheritance patterns have been suggested, including “2n + n” and “n + n” (Lalitha and Premachandran, 2007; Paterson et al., 2013). Studies suggest that higher-yielding varieties are the result of “2n+n” heredity (D'Hont et al., 1998). However, the limited chromosomal markers, gDNA probes, and repetitive probes hinders the further exploration of the cytogenetic mechanism underlying sugarcane nobilization.

Fluorescence *in situ* hybridization (FISH) is being applied in many biological research and has become an indispensable cytogenetic tool (Hu et al., 2014). Through the use of fluorescence, FISH allows the targeting and visualization of specific DNA within whole chromosomes or chromosomal regions (Younis et al., 2015). However, the exact individual chromosome identification is still challenging in plants due to a lack of chromosome-specific markers. Improvements in genomic sequencing have allowed the development of a novel FISH technique called oligonucleotide (oligo)-based FISH (Beliveau et al., 2012; de Oliveira Bustamante et al., 2021). In contrast to conventional FISH, oligo-FISH utilizes bioinformatics-designed probes, which has broadened the scope of cytogenetic researches (Schmutz et al., 2014; Han et al., 2015). Oligo-FISH has been used to study chromosomes and structural genomic variants in several crop species, including potato (Braz et al., 2018), maize (Braz et al., 2021), cucumber (Zhao et al., 2022), and rapeseed (Boideau et al., 2022) etc. Here, we applied oligo-based probes to identify the individual chromosomes of *S. officinarum* and *S. spontaneum*, and study the chromosomal heredity of their F_1_ hybrids. The results of this study provide a cytological basis for a better understanding of the nobilization in sugarcane breeding.

## 2 Materials and methods

### 2.1 Plant materials

Plant materials were obtained primarily from Fujian Agriculture and Forestry University and Yunnan Academy of Agricultural Sciences, China. The study materials, including *S. officinarum*, *S. spontaneum*, and their F_1_ hybrids, are reviewed in **Table 1**.

**Table 1:** The experimental plant materials used in this study.

Female parent	Species	Male parent	Species	F_1_ hybrids
Badila-CN	*S. officinarum*	Yunnan75-2-11	*S. spontaneum*	Yacheng82-108
Badila-CN	*S. officinarum*	Yacheng-spon	*S. spontaneum*	Yacheng58-43
Fiji	*S. officinarum*	Yacheng-spon	*S. spontaneum*	Yacheng75-4191
Yunnan Niuzhe	*S. officinarum*	Yacheng-spon	*S. spontaneum*	Yacheng75-409

### 2.2 Slide preparation

Root tips (~1 cm long) were pretreated with 1,4-dichlorobenzene-α-naphthylbromide for 2.5 h, then fixed with freshly-made Carnoy’s fixative (ethanol:glacial acetic acid, 3:1) for 24 h at 4 °C. The fixed samples were subsequently rinsed with distilled water and stored in 70% ethanol at -20°C. Root tips were digested with an enzymatic solution (1% pectolase Y-23, 2% pectolyase, 2% cellulase RS, 4% macerozyme R-10) for 3~4 h at 37°C, according to a previously-published protocol (Schwarzacher and Leitch, 1994). The digested root tip suspension was dropped on a slide and then an additional Carnot’s fixative was added to disperse the cells. Slide was used for microscopic examination and then the perfect metaphase cells were selected for further oligo-FISH.

### 2.3 Probe labeling and oligo-FISH

All oligo probe sequence libraries are available in Dataset S1(nph17905-sup-0001-Dataset1.xlsx) (Yu et al., 2022). Oligo probes were synthesized and labeled with either TAMARA- or FAM-, according to the method of Yu et al. (Yu et al., 2022). *S. spontaneum*-specific repetitive DNA probes (SsRetro1-SsRetro4) were directly-labeled with Cy3-dUTP, according to Huang et al. (Huang et al., 2020). Oligo-FISH was performed according to Braz et al. (Braz et al., 2020), with minor modifications. The treated slides were dehydrated in 75% and 100% ethanol sequentially for each 3 min. Briefly, slides were treated for 1.5 h with pepsin then fixed with 4% formaldehyde for 5 min and with 2× Saline Sodium Citrate (SSC) buffer for 3 min each. The treated slides were sequentially dehydrated with 75% and 100% ethanol for 3 min each. Next, the slides were denatured at 60°C for 90 s with 70% formamide (FD) and rapidly cooled to -20°C. The hybridization solutions (100% FD, 20 × SSC, 50% dextran sulphate (DS) and ~400 ng oligo probes) was added on the center of each slide. Hybridization was carried out at 37°C overnight. Next, the slides were washed using 2 × SSC for 5 min and 10 min, and twice with 1 × PBS for 5 min. The washed slides were then dried and counterstained with the 5 μg/mL 4′,6-diamidino-2- phenylindole (DAPI). Chromosome images were captured by Olympus BX53 camera epifluorescence microscope. The contrast of FISH images was adjusted and merged using Photoshop CS6 software.

## 3 Results

### 3.1 Individual *S. officinarum* chromosome identification using oligo-based painting

**Figure 1:**
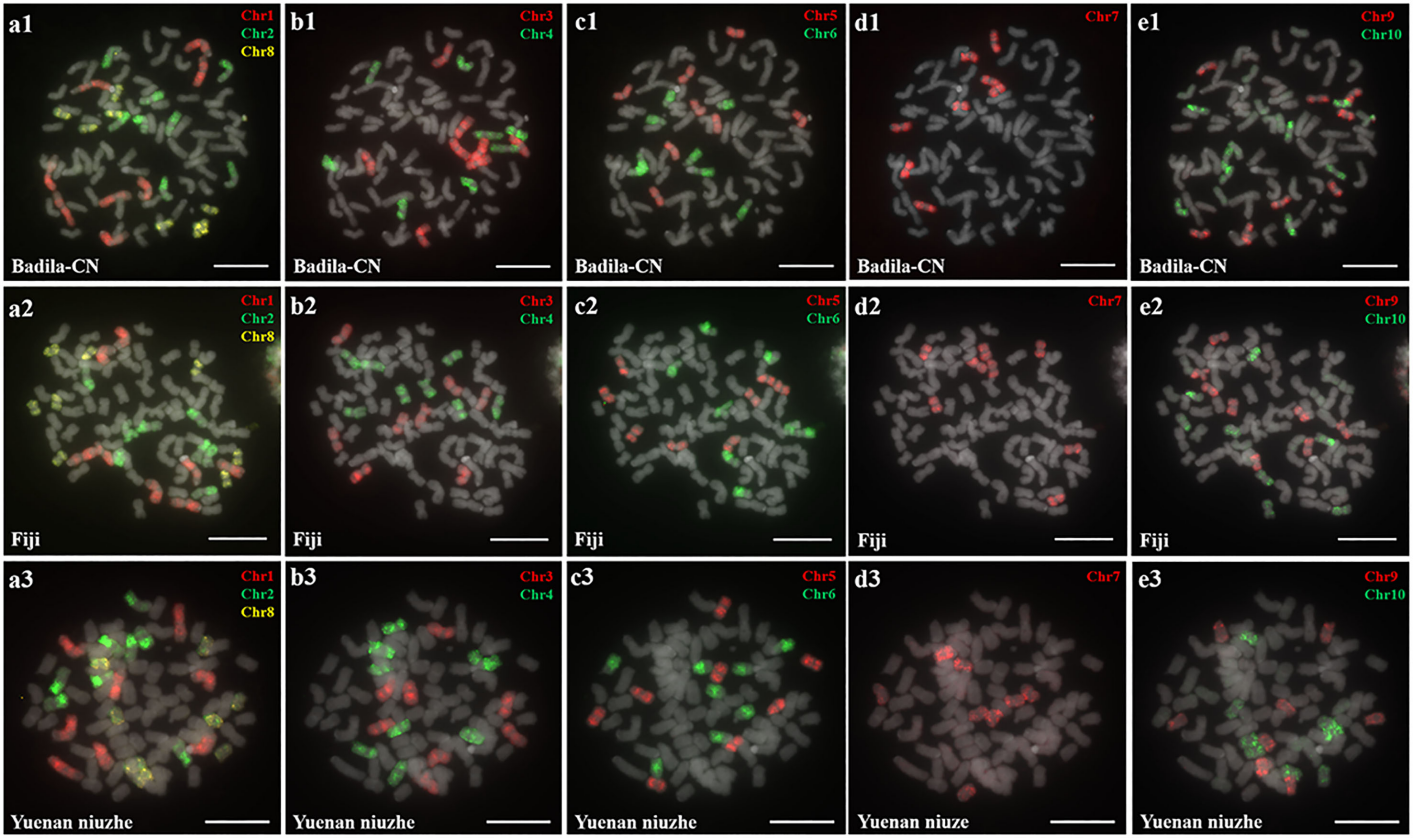
Metaphase chromosome identification of *S. officinarum*. **(A)** Chr1(red), Chr2 (green) and Chr8 (yellow); **(B)** Chr3 (red) and Chr4 (green); **(C)** Chr5 (red) and Chr6 (green); **(D)** Chr7 (red); **(E)** Chr9 (red) and Chr10 (green). Chromosome-specific probes were hybridized to somatic metaphase chromosomes prepared from Badila-CN **(A1–E1)**, Fiji **(A2–E2)**, and Yuenan niuzhe **(A3–E3)**. The gray chromosomes are counterstained by DAPI. Bars = 10 µm.

Oligo-based probes were designed based on the *S. officinarum* LA Purple genome and were anchored to chromosomes 1-10 (Yu et al., 2022). The ten chromosome-specific probes (Chr1-Chr10) were labeled using fluorescent dyes (TAMRA-, FAM-, or Cy5-) and hybridized to the somatic metaphase chromosomes prepared from three female *S. officinarum* parents (Badila-CN, Fiji and Yuenan Niuzhe) (**Figure 1**). Labeled Chr1-Chr10 probes will be expected to generate chromosome-specific fluorescence signals corresponding to chromosomes 1-10. For example, the Chr1, Chr2, and Chr8 painting probes, when hybridized to a metaphase cell, worked to identify chromosomes 1, 2, and 8, respectively (**Figure 1A1**). The slide was then washed prior to applying the Chr3 and Chr4 painting probes (**Figure 1B1**). All 10 chromosomes were successfully identified after five sequential rounds of FISH, and each probe hybridized to eight copies of a chromosome in Badila-CN (**Figures 1A1–E1**), Fiji (**Figures 1A2–E2**) and Yuenan Niuzhe (**Figures 1A3–E3**). These results indicate that all three *S. officinarum* have the same chromosomal composition and are autooctaploid with 2*n* = 8*x* = 80.

### 3.2 Individual *S. spontaneum* chromosome identification using oligo-based painting

**Figure 2:**
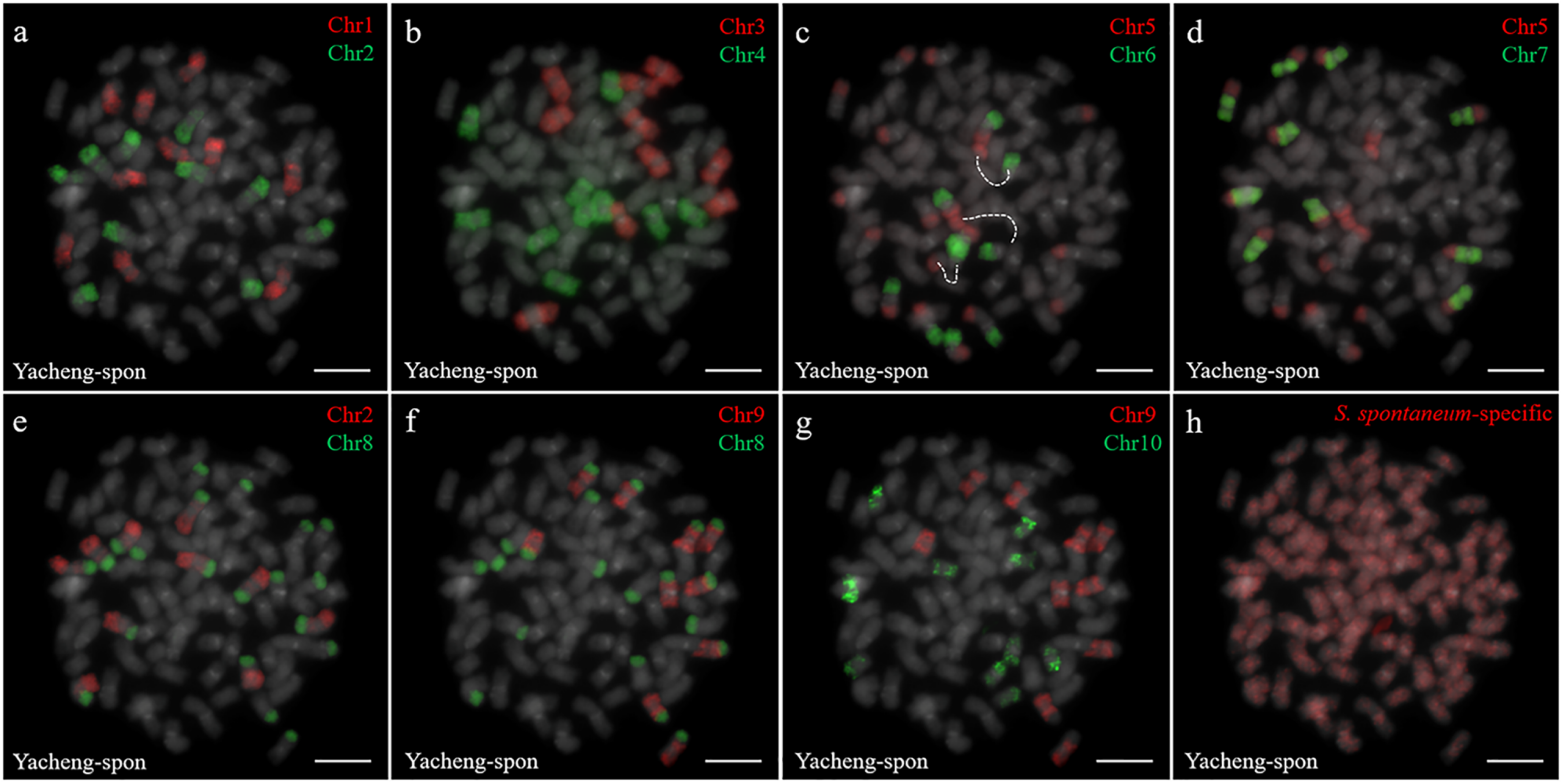
Metaphase chromosome identification of *S. spontaneum* Yacheng-spon. **(A)** Chr1(red) and Chr2 (green); **(B)** Chr3 (red) and Chr4 (green); **(C)** Chr5 (red) and Chr6 (green), with dotted lines linking the fusion chromosome SsChr5; **(D)** Chr5 (red) and Chr7 (green); **(E)** Chr2 (red) and Chr8 (green); **(F)** Chr9 (red) and Chr8 (green); **(G)** Chr9 (red) and Chr10 (green); **(H)** *S. spontaneum*-specific probe (red). The gray chromosomes are counterstained by DAPI. Bars = 10 µm.

**Figure 3:**
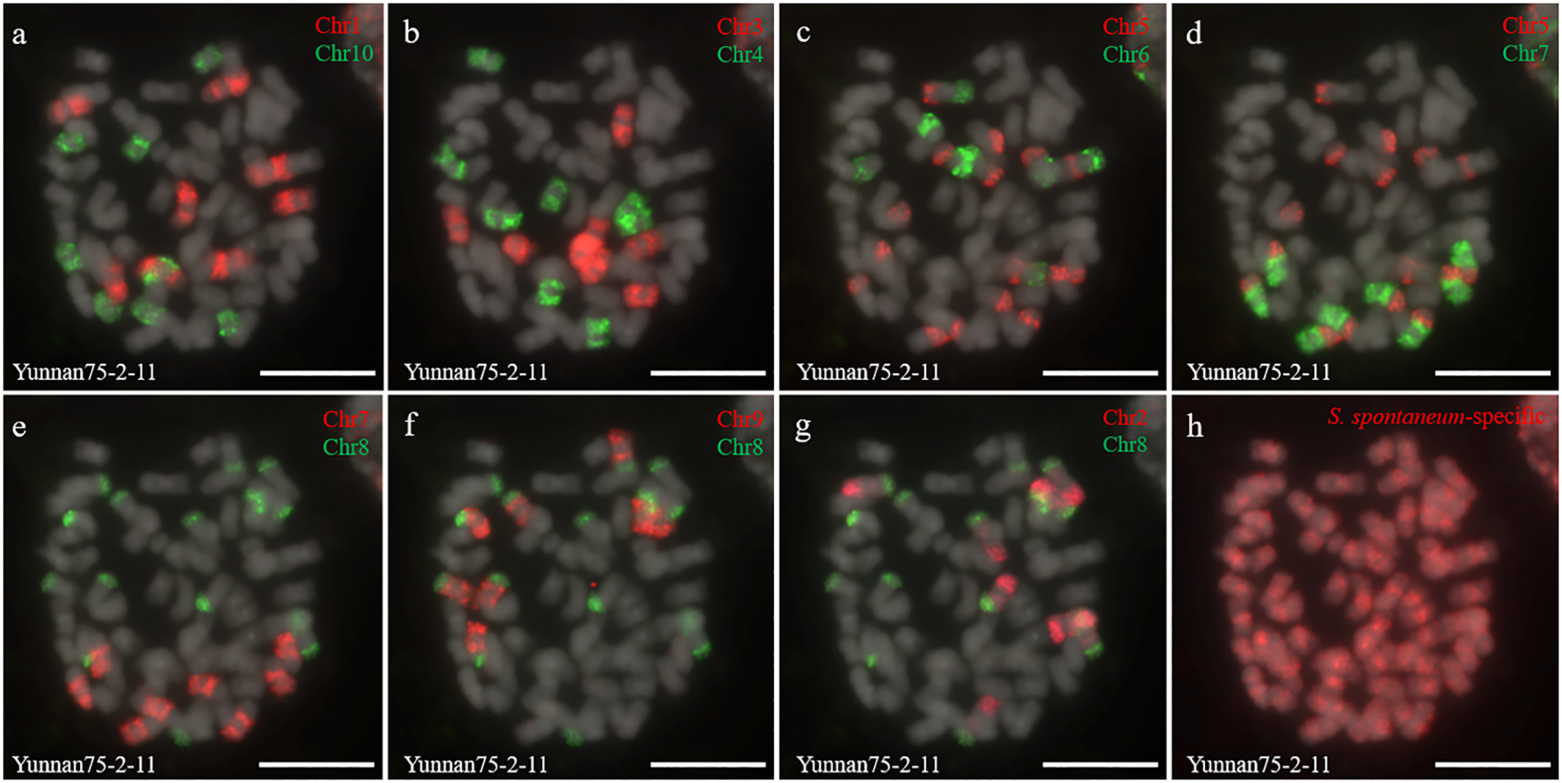
Metaphase chromosome identification of *S. spontaneum* Yunnan75-2-11. **(A)** Chr1(red) and Chr10 (green); **(B)** Chr3 (red) and Chr4 (green); **(C)** Chr5 (red) and Chr6 (green); **(D)** Chr5 (red) and Chr7 (green); **(E)** Chr7 (red) and Chr8 (green); **(F)** Chr9 (red) and Chr8 (green); **(G)** Chr2 (red) and Chr8 (green); **(H)** *S. spontaneum*-specific probe (red). The gray chromosomes are counterstained by DAPI. Bars = 10 µm.

Two *S. spontaneum* accessions, Yacheng-spon (2*n* = 80) and Yunnan75-2-11 (2*n* = 64), were used for individual chromosome analysis. Again, we used 10 *S. officinarum*-derived chromosome-specific painting probes hybridized to meiotic *S. spontaneum* chromosomes. Each of the 10 painting probes produced obvious FISH signals in both Yacheng-spon and Yunnan75-2-11. After six sequential rounds of chromosome painting, Yacheng-spon was confirmed to be a decaploid with a basic chromosome number *x* = 8 (**Figure 2**; **Table 2**). SsChr5 was hybridized with both the Chr5 and Chr6 probes, indicating that the *S. officinarum* 5-like chromosome was broken and fused with the *S. officinarum* 6-like chromosome to form SsChr5 of Yacheng-spon (**Figure 2C**), as well as the *S. officinarum* 7-like chromosome to form SsChr6 of Yacheng-spon (**Figure 2D**). Additionally, *S. officinarum* 8-like was broken and fused with *S. officinarum* 2-like and 9-like to form SsChr2 and SsChr7 of Yacheng-spon, respectively (**Figures 2E, F**). Individual chromosome of Yunnan75-2-11 were also identified, with similar fusions occurring in SsChr2, SsChr5, SsChr6 and SsChr7 (**Figure 3**). These results indicate that Yunnan75-2-11 is an octoploid with a basic chromosome number *x* = 8 (**Figure 3**; **Table 2**).

**Table 2:** Chromosomal composition of F_1_ hybrids between *S. officinarum* and *S. spontaneum*.

Chromosomes	Vegetative propagation								
	Badila-CN	Fiji	Yuenan niuzhe	Yacheng-spon	Yunnan75-2-11	Yacheng82-108	Yacheng58-43	Yacheng75-409	Yacheng75-4191
**SoChr1**	8	8	8	/	/	8	8	8	6
**SoChr2**	8	8	8	/	/	8	8	8	6
**SoChr3**	8	8	8	/	/	8	8	8	6
**SoChr4**	8	8	8	/	/	8	8	8	6
**SoChr5**	8	8	8	/	/	8	8	8	6
**SoChr6**	8	8	8	/	/	8	8	8	6
**SoChr7**	8	8	8	/	/	8	8	8	6
**SoChr8**	8	8	8	/	/	8	8	8	6
**SoChr9**	8	8	8	/	/	8	8	8	6
**SoChr10**	8	8	8	/	/	8	8	8	6
**SsChr1**	/	/	/	10	8	4	5	5	5
**SsChr2**	/	/	/	10	8	4	5	5	5
**SsChr3**	/	/	/	10	8	4	5	5	5
**SsChr4**	/	/	/	10	8	4	5	5	5
**SsChr5**	/	/	/	10	8	4	5	5	5
**SsChr6**	/	/	/	10	8	4	5	5	5
**SsChr7**	/	/	/	10	8	4	5	5	5
**SsChr8**	/	/	/	10	8	4	5	5	5
**Total**	80So	80So	80So	80Ss	64Ss	80So+32Ss	80So+40Ss	80So+40Ss	60So+40Ss

“So” indicates *S. officinarum* lineage. “Ss” indicates *S. spontaneum* lineage. “/” indicates not applicable.

### 3.3 Unveiling the individual chromosome inheritance of the F_1_ hybrids

The 10 chromosome-specific painting probes allow us essentially to identify each nonhomologous chromosomes in *S. officinarum* and *S. spontaneum*. Thus, we are interested in knowing how the individual chromosomes inheritance, as well as introgression of the *S. spontaneum* lineage into *S. officinarum*. Hence, four F_1_ hybrids between *S. officinarum* and *S. spontaneum*, Yacheng82-108, Yacheng58-43, Yacheng75-4191 and Yacheng75-409, were selected for chromosomal painting.

**Figure 4:**
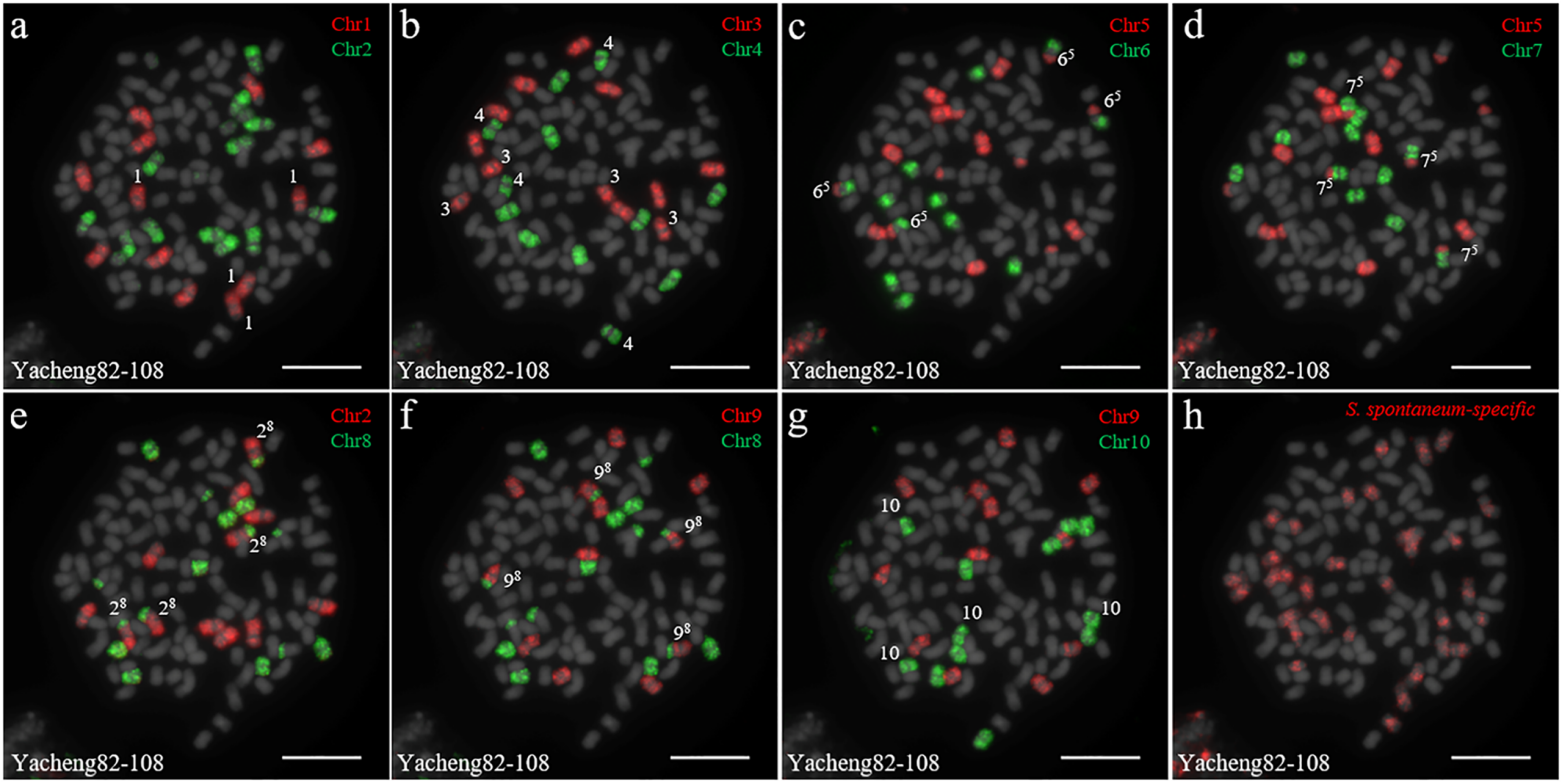
Chromosome identification of F_1_ hybrid Yacheng82-108. **(A)** Chr1(red) and Chr2 (green); **(B)** Chr3 (red) and Chr4 (green); **(C)** Chr5 (red) and Chr6 (green); **(D)** Chr5 (red) and Chr7 (green); **(E)** Chr2 (red) and Chr8 (green); **(F)** Chr9 (red) and Chr8 (green); **(G)** Chr9 (red) and Chr10 (green); **(H)** *S. spontaneum*-specific probe (red). The gray chromosomes are counterstained by DAPI. The Arabic numerals indicate *S. spontaneum* chromosomes. X^y^ indicates a fused chromosome in which chromosome Y was broken and fused with chromosome X. Bars = 10 µm.

We first identified all chromosomes through multiple rounds of oligo-FISH, and then distinguished *S. spontaneum*-specific chromosomes using *S. spontaneum*-specific probes applied to the same cell. In Yacheng82-108 (Badila-CN × Yunnan75-2-11), each of the 10 oligo probes generated symmetrical signals through multiple rounds of FISH (**Figure 4**). The chromosomal colocalization results indicated that Yacheng82-108 contains a total of 112 chromosomes, including 80 derived from *S. officinarum* and 32 derived from *S. spontaneum* (**Figure 4**; **Table 2**). Each set of nonhomologous chromosomes was uniformly inherited from their parents.

**Figure 5:**
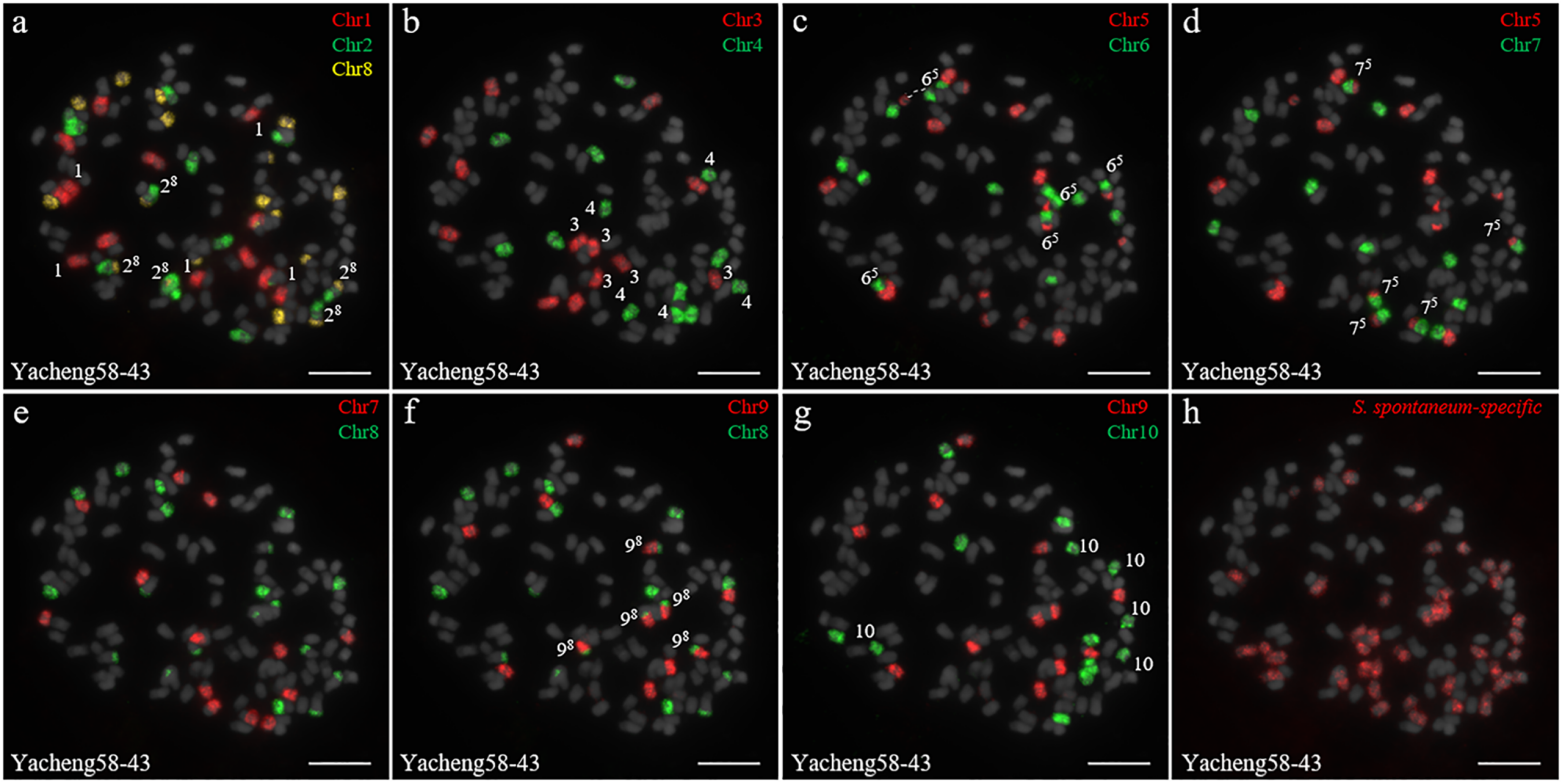
Chromosome identification of F_1_ hybrid Yacheng58-43. **(A)** Chr1(red) Chr2 (green) and Chr8 (yellow); **(B)** Chr3 (red) and Chr4 (green); **(C)** Chr5 (red) and Chr6 (green), with dotted lines linking the fusion chromosome SsChr5; **(D)** Chr5 (red) and Chr7 (green); **(E)** Chr7 (red) and Chr8 (green); **(F)** Chr9 (red) and Chr8 (green); **(G)** Chr9 (red) and Chr10 (green); **(H)** *S. spontaneum*-specific probe (red). The gray chromosomes are counterstained by DAPI. The Arabic numerals indicate *S. spontaneum* chromosomes. X^y^ indicates a fused chromosome in which chromosome Y was broken and fused with chromosome X. Bars = 10 µm.

**Figure 6:**
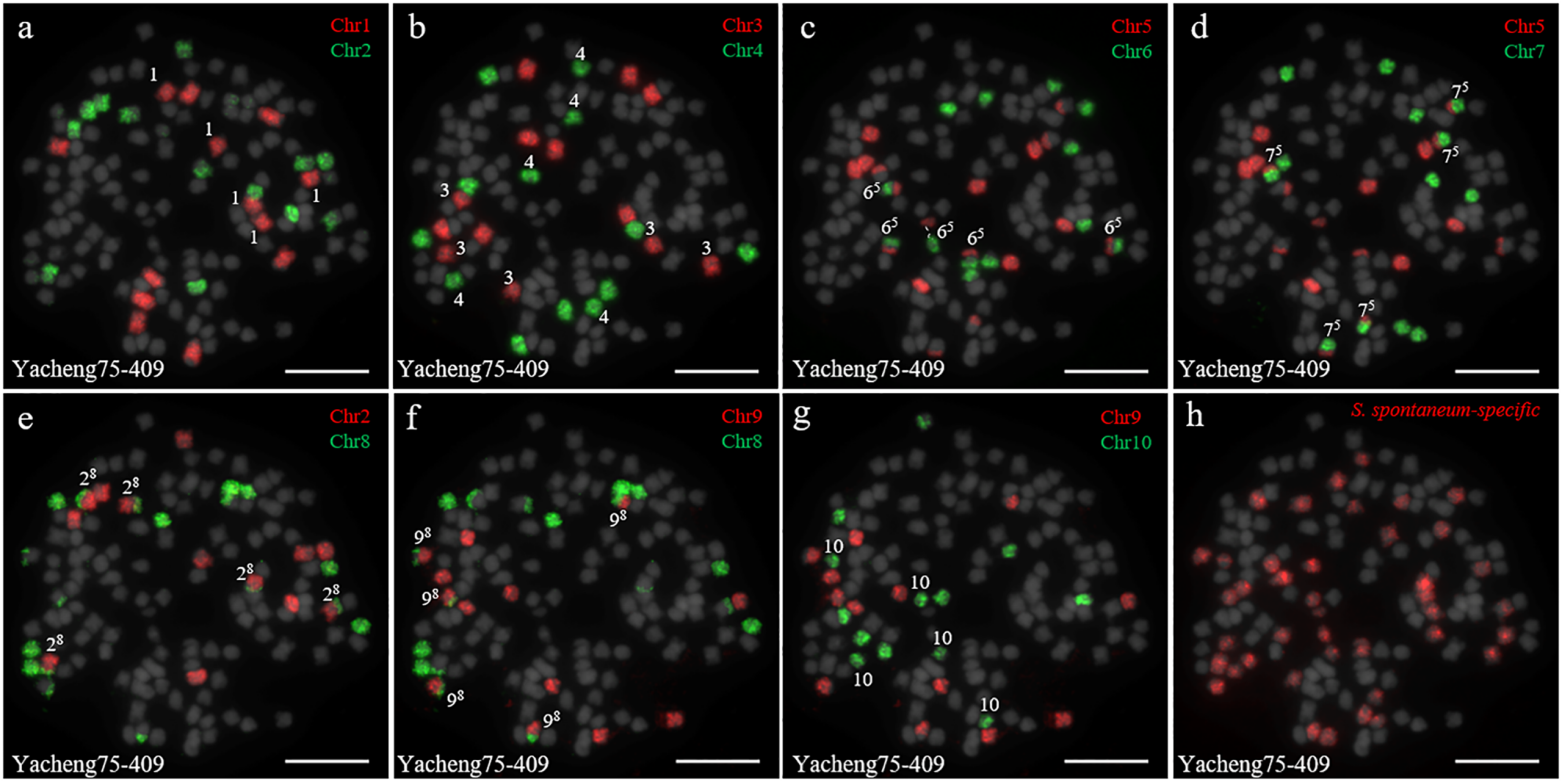
Chromosome identification of F_1_ hybrid Yacheng75-409. **(A)** Chr1(red) and Chr2 (green); **(B)** Chr3 (red) and Chr4 (green); **(C)** Chr5 (red) and Chr6 (green), with dotted lines linking the fusion chromosome SsChr5; **(D)** Chr5 (red) and Chr7 (green); **(E)** Chr2 (red) and Chr8 (green); **(F)** Chr9 (red) and Chr8 (green); **(G)** Chr9 (red) and Chr10 (green); **(H)** *S. spontaneum*-specific probe (red). The gray chromosomes are counterstained by DAPI. The Arabic numerals indicate *S. spontaneum* chromosomes. X^y^ indicates a fused chromosome in which chromosome Y was broken and fused with chromosome X. Bars = 10 µm.

**Figure 7:**
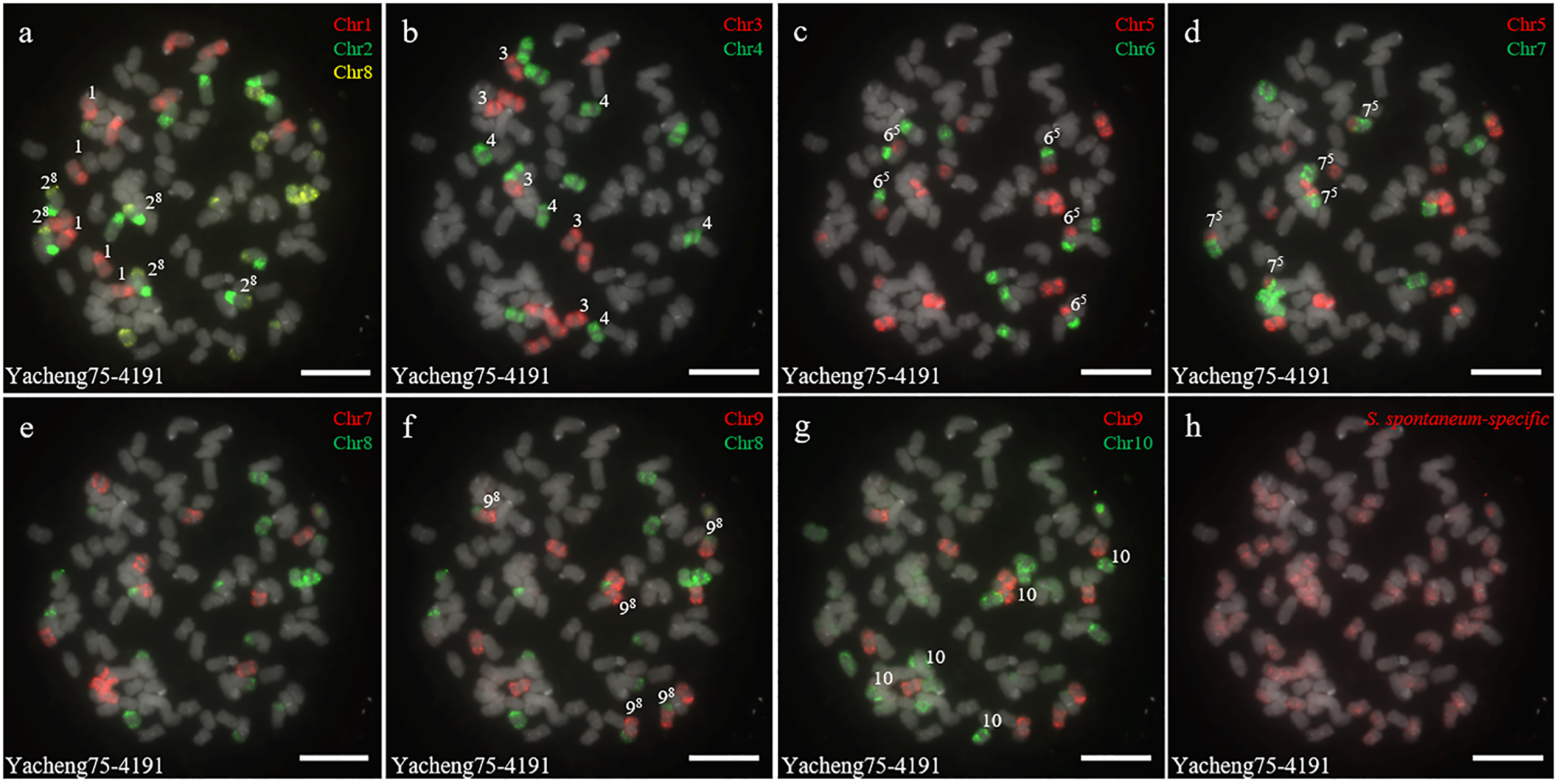
Chromosome identification of F_1_ hybrid Yacheng75-4191. **(A)** Chr1(red) Chr2 (green) and Chr8 (yellow); **(B)** Chr3 (red) and Chr4 (green); **(C)** Chr5 (red) and Chr6 (green); **(D)** Chr5 (red) and Chr7 (green); **(E)** Chr7 (red) and Chr8 (green); **(F)** Chr9 (red) and Chr8 (green); **(G)** Chr9 (red) and Chr10 (green); **(H)** *S. spontaneum*-specific probe (red). The gray chromosomes are counterstained by DAPI. The Arabic numerals indicate *S. spontaneum* chromosomes. X^y^ indicates a fused chromosome in which chromosome Y was broken and fused with chromosome X. Bars = 10 µm.

In addition, we chose three other F_1_ hybrids from the same male parent Yacheng-spon but with different female *S. officinarum* parents. For Yacheng58-43, the painting probes generated distinct signals (**Figure 5**). FISH indicated that Yacheng58-43 (2*n*) contained 120 somatic chromosomes, including 40 derived from *S. spontaneum* chromosomes (in which half of each chromosome was derived from the male parent) and 80 derived from *S. officinarum*. The same chromosomal composition was also found for Yacheng75-409 (**Figure 6**). Yacheng75-4191 contained 40 chromosomes derived from *S. spontaneum* (**Figure 7**, labeled with Arabic numerals), but a different number of chromosomes derived from *S. officinarum*. Each of the 10 painting probes hybridized to six *S. officinarum* chromosomes in Yacheng75-4191, suggesting that 1.5n of female parent (*S. officinarum*) chromosomes were transmitted to Yacheng75-4191 (**Figure 7**; **Table 2**). Finally, FISH results indicated that the chromosomal transmission of *S. spontaneum* is “n” in all F_1_ hybrids.

## 4 Discussion

Identification of individual chromosome is essential to understand chromosome organization and evolution of related species (Jiang and Gill, 2006; Xiong and Pires, 2011). In plants, chromosome painting with oligo-based probes has been used for karyotyping as well as studies of chromosomal rearrangement, meiotic pairing and recombination (Jiang, 2019). Sugarcane has one of the most complex genetic backgrounds, and exhibits high degrees of polyploidy as well as aneuploidy (Heinz et al., 1969). The use of chromosome-specific barcodes and painting probes has revealed the complex chromosomal structure and evolutionary history of sugarcane and related species (Meng et al., 2020; Meng et al., 2022; Yu et al., 2022). Sugar-rich *S. officinarum* typically has either 2*n* = 80 or 81 chromosomes, with plants containing more than 81 chromosomes likely to be hybrids (Bremer, 1961; Piperidis et al., 2010; Piperidis and D’Hont, 2020). In this study, we found that *S. officinarum* Fiji and Yuenan niuzhe each possessed eight copies of homologous chromosomes, suggesting that they are typical octaploids (**Figure 1**). These results support the classical view that *S. officinarum* is characterized as an autooctaploid with *x* = 10 (**Figure 1**). For *S. spontaneum*, previous cytological evidence indicated that the *x* = 8 cytotype was derived from *x* = 10 through consecutive chromosomal breaking and fusion. In this study we used chromosome-specific paints based on the *S. officinarum* genome and found that both *S. spontaneum* Yunnan75-2-11 and Yacheng-spon were *x* = 8. Consistent with previous results, we also found that the *S. officinarum* 5-like and *S. officinarum* 8-like chromosomes had been broken and fused. Additionally, we found that SsChr5 is most susceptible to breakage at the *S. officinarum-*like chromosome 5-6 fusion point in *S. spontaneum* Yacheng-spon (**Figure 2**). Notably, this phenomenon is genetically transmitted to the offspring (**Figures 5**, **6**), potentially leading to meiotic instability.

*S. spontaneum* has been widely used for improving sugarcane genetics due to its excellent agronomic characteristics and the increasing demand for stress-resistant sugarcane cultivars (De Morais et al., 2015; Racedo et al., 2016). In nobilization, the retention of *S. spontaneum*-derived resistance genes is accompanied by the presence of unreduced *S. officinarum* gametes, leading to high sugar content (Lu et al., 1994). The varied cytotypes and rich genetic diversity of *S. spontaneum* have hampered cytogenetic studies in sugarcane (Zhang et al., 2018). Previous studies suggest that either “2n + n” or “n + n” transmission occurs in the F_1_ hybrids between *S. officinarum* and *S. spontaneum* (Price, 1957; Piperidis et al., 2010). However, the available cytogenetic probes, 5S rDNA, 35S rDNA, and genomic DNA, have been unable to identify all non-homologous chromosomes in these hybrids. Chromosomal inheritance in F_1_ hybrids between *S. officinarum* and *S. spontaneum* was preliminarily studied based on chromosome numbers (D'Hont et al., 1998; Yu et al., 2018). However, the precise mechanism of individual chromosome transmission remained largely unknown. In this study, we combined chromosome-specific oligo-based probes and *S. spontaneum*-specific repeat probes to identify all ten sugarcane chromosomes and determine whether they were derived from *S. officinarum* or *S. spontaneum* (**Figures 4** **–** **7**). Our system proved highly efficient for tracing the precise chromosomal inheritance pattern in hybrids of *S. officinarum* and *S. spontaneum*, and will aid in efforts to further utilize *S. spontaneum* in sugarcane breeding.

Based on nobilization theory, sugarcane breeders have carried out many researches for improving the genetic background of sugarcane. However, chromosome inheritance mechanism of *S. officinarum* is still confused and dubious in the hybrids between *S. officinarum* and *S. spontaneum*. Previously, “2n + n” chromosome transmission has been found by the method of only using genomic *in situ* hybridization, but this technique cannot verify whether the *S. officinarum* chromosomes were fully doubled (Price, 1957; Piperidis et al., 2010; Yu et al., 2018). In this study, by combining *S. officinarum*-derived chromosome paints and *S. spontaneum*-specific probes, we that all three studied F_1_ hybrids (Yacheng82-108, Yacheng58-43, Yacheng75-409) had “2n + n” chromosome transmission (**Figures 4** **–** **6**). These results suggest that each non-homologous *S. officinarum* chromosomes was completely doubled. Interestingly, we found one F_1_ hybrid (Yacheng75-4191) exhibited “1.5n + n” transmission, suggesting that 1.5 × of *S. officinarum* chromosomes were inherited (**Figure 7**). Altogether, these results will broaden our understanding of the sugarcane nobilization between *S. officinarum* and *S. spontaneum*. Furthermore, these chromosome-specific identification results will be useful for detailed physical mapping for use in guiding breeders to select suitable parents for sugarcane breeding.

## 5 Conclusion

In the present study, the chromosome-specific painting probes derived from *S. officinarum* can be used for accurately identifying individual chromosome and chromosomal heredity of F_1_ hybrids during sugarcane nobilization. We discovered that nonhomologous *S. officinarum* chromosomes were completely doubling in most F_1_ hybrids. However, the F_1_ hybrid Yacheng75-4191 exhibited defective chromosome doubling with 1.5n of *S. officinarum* chromosomes transmission. These results support previous genetic studies of *S. spontaneum* and provide more useful molecular cytogenetic data for its F_1_ hybrids. In addition, these results provide robust chromosome markers for in-depth studies into the molecular mechanism underlying chromosome doubling during the nobilization, as well as tracing chromosomal inheritance for sugarcane breeding.

## Data availability statement

The original contributions presented in the study are included in the article/supplementary material. Further inquiries can be directed to the corresponding authors.

## Author contributions

ZD and FY designed the research. JC, LX and JL performed the experiments. WY provided the plant resources. MZ, ZD, and FY analyzed the results and wrote the manuscript. All authors contributed to the article and approved the submitted version.
